# COVID‐19—The impact of variable and “low normal” pulse oximetry scores on Oximetry@Home services and clinical pathways: Confounding variables?

**DOI:** 10.1002/nop2.957

**Published:** 2021-06-23

**Authors:** Nicholas Harland, Jane Greaves, Elizabeth Fuller

**Affiliations:** ^1^ Faculty of Health Science and Wellbeing Helen McArdle Nursing and Care Research Institute University of Sunderland Sunderland UK; ^2^ Northumbria University Newcastle upon Tyne UK; ^3^ South Tyneside NHS Foundation Trust South Shields UK

**Keywords:** COVID‐19, pulse oximeter, oxygen saturation, oximetry@home

## Abstract

COVID‐19 Oximetry@Home services have been commissioned nationally. This allows higher‐risk patients with mild COVID‐19 symptoms to remain at home, being supplied with a Pulse Oximeter to measure their oxygen saturation (SpO_2_) two to three times daily for two weeks. Patients record their readings manually or electronically which are monitored by a clinical team. Clinical decisions, using an algorithm, are based on SpO_2_ readings in a narrow range with 1–2 point changes potentially affecting care. In this article, we discussed the problem that multiple factors affect SpO_2_ readings, and that some “normal” individuals will have “low‐normal” scores at the threshold of clinical management, without any known respiratory problem. We discuss the potential magnitude of this problem based on the associated literature and consider how this will have an impact on the use of the Oximetry@home services, potentially partially confounding their purpose; to reduce face‐to‐face medical care.

## 1 BACKGROUND

There are a number of advantages in managing less‐serious cases of COVID‐19 in the community, though this limits, the use of medical devices such as thermometers, stethoscopes and pulse oximeters during the assessment. With the usefulness of patient performed home‐based pulse oximetry in both preventing unnecessary emergency department attendance (Torjesen, 2020) and in early identification of silent hypoxia, however, NHS England has recommended national commissioning of “Oximetry@Home” services (NHSE, 2020a) where patients with mild COVID‐19 symptoms but at higher risk of deterioration can be given with a Pulse Oximeter for 14 days in order to self‐monitor their oxygen saturation (SpO_2_) 2–3 times a day.

Patients referred to Oximetry@Home services are usually directed to use an App or a paper diary to record their observations. The App either gives an automated response/recommendation, or data are monitored by a clinician who can contact the patient if necessary, though usually only during normal working hours. Patients are given instructions on interpreting their results so they can act independently if they need to, such as seeking urgent care. Those aged over 65 and/or with multiple comorbidities defining them as extremely vulnerable are being targeted by the pathway due to a higher risk of deterioration (NHSE, 2020a).

The assessment of patients in Oximetry@Home services starts with their oxygen saturation as measured by a Pulse Oximeter, SpO_2_, followed by consideration of other signs and symptoms. A Red, Amber, Green (RAG) rating is used and patients are classified as Red if their SpO_2_ is 92% or less, Amber if their SpO_2_ is 93% or 94%, and Green if their SpO_2_ is 95% or greater. Usually, only Green patients are eligible for Oximetry@Home (NHSE, 2020b). Various non‐disease related factors influence SpO_2_ scores, however, and these may not be accounted for in the pathway. In this article, we discuss the impact of various factors affecting SpO_2_ may have on the movement of patients in and out of Oximetry@Home services that may partially confound their purpose to ease the pressure on face‐to‐face medical services.

## 2 THE VARIABILITY OF OXYGEN SATURATION MEASUREMENTS

The accepted range of “normal” for oxygen saturation in the blood as measured by a pulse oximeter (SpO_2_) is 95%–99%. This statement is so ubiquitous that medical articles rarely reference it, though documents such as the World Health Organisation Pulse Oximetry Training Manual exist (WHO, 2011). When searching for normative data about SpO_2_ in a non‐medical population, little information can be found. In a study of 791 individuals aged 65 and over (Rodríguez‐Molinero et al., 2013), the mean 5th centile SpO_2_ score was 92% after accounting for variables such as COPD, indicating that 5% of the measured population had a significantly low score without any known medical explanation. In another study of 458 individuals aged between 40–79 (Enright & Sherrill, 1998), the range of oxygen saturation before a 6‐min walk test was 92%–98% at the 5th centile and 93%–99% at the 95th centile. Neither study documented in detail the procedure used to measure SpO_2_.

A population study of 5,152 individuals in Norway (Vold et al., 2015) found that 11.5% had a low, or low end of normal, SpO_2_ of less than or equal to 95%. In this study, only a minority of the individuals with a low SpO_2_ were reported to have asthma (18%) or COPD (13%), whereas a statistically significant majority had a BMI over 25 (77%), and a large proportion was aged 70 or over (46%). In the United Kingdom, 24.4% of cases tested for COVID‐19 between May–August 2020 were aged 60 or over and 15% were aged 70 or over [8] (Department of Health & Social Care, 2020). Although the Norwegian study suggests 11.5% of any population may have a low SpO_2_, most of these cases having no known respiratory diagnosis, the literature suggests there may be “missing millions” with undiagnosed COPD (Bakerly & Cardwell, 2016) and a potentially high rate of undiagnosed Obesity Hypoventilation Syndrome (Masa et al., 2019). A statistically significant proportion of unexplained “low normal” SpO_2_ scores found in population studies may have an undiagnosed respiratory condition.

In addition to population variance, specific factors about the protocol used to measure SpO_2_ may affect the outcome. Measurements taken whilst lying at rest are statistically significantly different from those taken in sitting (Ceylan et al., 2015). Further to this, and age and obesity factors, SpO_2_ may drop over a period of 5–15 min at rest (Mehta & Parmar, 2017) and more specifically at rest during meditation (Bernardi et al., 2017). Limb temperature, associated with ambient temperature, may also have a statistically significant effect (Khan et al., 2015) as can anxiety, the presence of which may drop scores a full point (Ardaa et al., 2020). Lastly, it is known that Pulse Oximeters have a standard error of measurement of ± 2% when compared to simultaneous arterial blood gas measurement, SaO_2_, (American Thoracic Society, 2018) but pragmatically, from a clinical perspective, as there is no way to account for this variance measurements must be taken and acted upon at face value.

Variability of SpO_2_ over time and repeated measurement is a further issue, with little information about this in a non‐medical population. One study with a small sample size (*n* = 36) examined SpO_2_ changes over an hour [16] (Bhogal & Mani, 2017) but no reports exist of variability during repeated measurement over weeks, as undertaken during Oximetry@Home.

During a 14 day Oximetry@Home monitoring period with SpO_2_ taken three times a day, and potentially more frequently in an anxious patient, 42 measurements could be taken. Even assuming an identical measurement protocol is used on every occasion and a stable clinical condition, it is reasonable to suggest there will be some variability in these measurements. With the population research using one measurement taken on one occasion showing that 11.5% of individuals may have an SpO_2_ of 95% or below, the probability of finding a low reading on one or more occasions during repeated measurement over time when following the COVID‐19 recommendations is probably higher than 11.5%.

## 3 THE IMPACT OF SpO2 VARIABILITY ON THE OXIMETRY@HOME PATHWAY

The algorithm behind Oximetry@Home services recommends that because poor outcomes are associated with lower SpO_2_ scores [17] (Shah et al., 2020); those whose SpO_2_ drops to 93%–94% should receive face‐to‐face medical assessment and be considered for hospital admission, and those with scores of 92% or below should receive urgent secondary medical care. With nationwide implementation of Oximetry@Home services, repeated SpO_2_ measurements taken at home by patients will be an important factor in interpreting their clinical condition.

SpO_2_ measurement is most frequently performed in a short time of oximeter placement with the patient in sitting and not having had a period of rest, walking from a waiting area to a clinical area physiologically interrupting rest. With commissioning of Oximetry@Home services an NHS YouTube video (2020) has been released that recommends for home‐based measurement patients lie down for 5 min, place the Oximeter, and then take the most stable reading 1 min after placement. This video link has been circulated via the Future NHS Collaboration Platform pages relevant to those setting up Oximetry@Home services but no account appears to have been made about the potentially lower reading this can give compared to readings taken in sitting. It is noteworthy that a further Health Education England NHS video featured in the Daily Mail newspaper recommends an entirely different protocol, taking the first reading given in sitting (Daily Mail, 2020).

In an individual with an unknown usually, low score of 95%, a drop of even 1 point due to COVID‐19 infection could result in an Amber rating leading to direct clinical care. What is unclear is whether a single point drop in an individual with a pre‐disease low score makes direct clinical care an efficient use of resources.

Although the national algorithm also mentions SpO_2_ drop, with the vast majority of cases not having documented pre‐disease SpO_2_ scores this factor becomes impossible to assess until after any initial drop caused by the virus that led to SpO_2_ assessment. It is also unclear clinically from a decision making perspective if an individual's best saturation/perfusion level in sitting should be the baseline around which care is organised, or if a reduced saturation/perfusion in lying after rest should be the baseline. No nationally agreed policy about this appears to exist.

## 4 DISCUSSION

SpO_2_% is an eye‐catching, publicly available parameter in the evaluation of COVID‐19. NHS England has acquired 370,000 oximeters for multi‐patient use for distribution to services.

It is probable that the factors described may result in many single point SpO_2_ measurement changes triggering face‐to‐face patient reviews either in primary care or emergency departments. Over time many thousands of patients may be treated in the community with SpO_2_ monitoring, potentially leading to a statistically significant number of unnecessary face‐to‐face reviews. When the effect of factors affecting SpO_2_ readings in COVID‐19 cases where no pre‐disease SpO_2_ readings are available are analysed and placed into the context of population‐based clinical and home‐based measurement, the potential impact is statistically significant, particularly on those “missing millions” more likely to have borderline SpO_2_. Additionally, Oximetry@Home services are far more likely to select those with borderline scores by targeting the over 65’s and those that may have a higher BMI associate with comorbidities. The research suggests the “low normal” population will be at least 11.5% of all individuals, but due to the selection criteria of Oximetry@Home services, this percentage appears probably to be much greater than this.

With the documented factors affecting SpO_2_ scores at play, those patients with usually lower scores, particularly scores of 95%, may potentially move between green and amber ratings on multiple occasions. This move could possibly even occur between usual clinical practice measurement in sitting at the time of referral to Oximetry@Home, and a patients’ first measure at home if they use the lying down for 6 min protocol. Patient anxiety upon taking a measurement if they feel unwell could also potentially move those with a borderline score to drop below 95% and seek care. This could cause multiple unnecessary episodes of face‐to‐face care putting additional pressure on services already working at, or beyond, capacity.

Even outside commissioned Oximetry@Home pathways and medical supply of oximeters to patients, press coverage of the usefulness of pulse oximeters has been widespread and it is unknown what percentage of the population may own a pulse oximeter in response to the COVID‐19 Pandemic, though with many different suppliers of relatively inexpensive devices and reports of devices selling out (CNN, 2020) the number is likely to be in the hundreds of thousands at least. The factors described in this article may also affect these individuals, putting further pressure on services.

## 5 RECOMMENDATIONS

1. Due to the device measurement error inherent in pulse oximeters and the narrow boundaries of the national oximetry@home algorithm it is recommended that, where possible, the mean of three measurements should be used to inform patient and/or clinical decision‐making. Patients should be advised that upon taking a reading that might cause them to seek medical care, gave their condition is not obviously subjectively deteriorating, they should repeat the reading three times over a period of one hour before making a decision.
2. Unification and documentation of SpO_2_ measurement protocols with a single approved national measurement protocol put in place for both patients and clinicians. Pragmatically in an outpatient and home setting this protocol should probably be in the sitting position.
3. Widespread clinical education of relevant NHS staff about the potential for low scores and the factors that increase the likelihood of those scores to facilitate a more nuanced approach to implementation of the national algorithm.

## CONFLICT OF INTEREST

None of the authors has any conflict of interest to declare.

## AUTHOR CONTRIBUTIONS

We declare that each of the listed authors has substantively contributed to the production of this article, contributing to both ideas and written content.

## ETHICAL APPROVAL

As an analysis of the literature Research Ethics Committee Approval is not applicable to this article submission.

## PATIENT CONSENT STATEMENT

As an analysis of the literature patient consent is not applicable to this article submission.

## Data Availability

Data sharing not applicable to this article as no datasets were generated or analysed during the current study.

## References

[nop2957-bib-0001] American Thoracic Society Patient Education Series . (2018). Pulse oximetry. American Journal of Respiratory and Critical Care Medicine, 184, 1.

[nop2957-bib-0002] Ardaa, K. N. , Akay, S. , & Yetkin, S. (2020). Is there a relationship between oxygen saturation and MRI‐induced anxiety? A prospective study. Clinical Imaging, 60, 147–152. 10.1016/j.clinimag.2019.12.005 31927169

[nop2957-bib-0003] Bakerly, N. D. , & Cardwell, G. (2016). The ‘missing millions’ Where do we find them? Chronic Respiratory Disease, 13, 319–320. 10.1177/1479972316667362 30209971 PMC5734806

[nop2957-bib-0004] Bernardi, F. , Bordino, M. , Bianchi, L. , & Bernardi, L. (2017). Acute fall and long‐term rise in oxygen saturation in response to meditation. Psychophysiology, 54, 1951–1966. 10.1111/psyp.1297 28840941

[nop2957-bib-0005] Bhogal, A. S. , & Mani, A. R. (2017). Pattern analysis of oxygen saturation variability in healthy individuals: Entropy of pulse oximetry signals carries information about mean oxygen saturation. Frontiers in Physiology, 8, 555. 10.3389/fphys.2017.00555 28824451 PMC5539125

[nop2957-bib-0006] Ceylan, B. , Khorshid, L. , Gunes, U. Y. , & Ayten, Z. A. (2015). Evaluation of oxygen saturation values in different body positions in healthy individuals. Journal of Clinical Nursing, 25, 1095–1100. 10.1111/jocn.13189 26879626

[nop2957-bib-0007] CNN online . (2020, April 26). People are buying pulse oximeters to try and detect coronavirus at home. Do you need one? Retrieved from https://edition.cnn.com/2020/04/26/health/pulse‐oximeters‐coronavirus‐wellness‐scn‐trnd/index.html

[nop2957-bib-0008] Daily Mail . (2020, November 10). Retrieved from https://www.dailymail.co.uk/news/article‐8933117/Covid‐19‐patients‐slight‐drop‐oxygen‐levels‐risk‐death.html

[nop2957-bib-0009] Department of Health and Social Care . (2020). Demographic data for coronavirus (COVID‐19) testing (England): 28 May to 26 August. Retrieved from https://www.gov.uk/government/publications/demographic‐data‐for‐coronavirus‐testing‐england‐28‐may‐to‐26‐august/demographic‐data‐for‐coronavirus‐covid‐19‐testing‐england‐28‐may‐to‐26‐august#table‐3

[nop2957-bib-0010] Enright, P. L. , & Sherrill, D. L. (1998). Reference equations for the six‐minute walk in healthy adults. American Journal of Respiratory and Critical Care Medicine, 158, 1384–1387. 10.1164/ajrccm.158.5.9710086 9817683

[nop2957-bib-0011] Health Education England . (2020, November 4) Retrieved from https://www.youtube.com/watch?v=ifnYjD4IKus&feature=youtu.be

[nop2957-bib-0012] Khan, M. , Pretty, C. G. , Amies, A. C. , Elliot, R. , Shaw, G. M. , & Chase, G. (2015). Investigating the effects of temperature on photoplethysmography. IFAC‐PapersOnLine, 48, 360–365. 10.1016/j.ifacol.2015.10.166

[nop2957-bib-0013] Masa, J. F. , Pépin, J. L. , Borel, J. C. , Mokhlesi, B. , Murphy, P. B. , & Sánchez‐Quiroga, M. A. (2019). Obesity hypoventilation syndrome. European Respiratory Review, 28, 180097. 10.1183/16000617.0097-2018 30872398 PMC9491327

[nop2957-bib-0014] Mehta, J. N. , & Parmar, L. D. (2017). The effect of positional changes on oxygenation in patients with head injury in the intensive care unit. Journal of Family Medicine and Primary Care, 6, 853–858. 10.4103/jfmpc.jfmpc_27_17 PMC5848412 29564277

[nop2957-bib-0015] NHS England . (2020a). Novel coronavirus (COVID‐19) standard operating procedure – COVID Oximetry @home. NHS England. Retrieved from https://www.england.nhs.uk/coronavirus/wp‐content/uploads/sites/52/2020/06/C0445‐remote‐monitoring‐in‐primary‐care‐revised.pdf

[nop2957-bib-0016] NHS England . (2020b). Pulse oximetry to detect early deterioration of patients with COVID‐19 in primary and community care settings. NHS England. https://www.england.nhs.uk/coronavirus/wp‐content/uploads/sites/52/2020/06/C0445‐remote‐monitoring‐in‐primary‐care‐revised.pdf

[nop2957-bib-0017] Rodríguez‐Molinero, A. , Narvaiza, L. , Ruiz, J. , & Gálvez‐Barrón, C. (2013). Letters to the Editor, Normal respiratory rate and peripheral blood oxygen saturation in the elderly population. Journal of the American Geriatrics Society, 61, 2238–2240. 10.1111/jgs.12580 24329828

[nop2957-bib-0018] Shah, S. , Majmudar, K. , Stein, A. , Gupta, N. , Suppes, S. , Karamanis, M. , Capannari, J. , Sethi, S. , & Patte, C. (2020). Novel use of home pulse oximetry monitoring in COVID‐19 patients discharged from the emergency department identifies need for hospitalization. Academic Emergency Medicine, 27, 681–692. 10.1111/acem.14053 32779828 PMC7323027

[nop2957-bib-0019] Torjesen, I. (2020). Covid‐19: Patients to use pulse oximetry at home to spot deterioration. BMJ, 371, m4151. 10.1136/bmj.m4151

[nop2957-bib-0020] Vold, M. L. , Ulf Aasebø, U. , Wilsgaard, T. , & Melbye, H. (2015). Low oxygen saturation and mortality in an adult cohort: The Tromsø study. BMC Pulmonary Medicine, 15, 9. 10.1186/s12890-015-0003-5 25885261 PMC4342789

[nop2957-bib-0021] World Health Organisation . (2011). Patient safety. Pulse Oximetry training manual. Retrieved from https://www.who.int/patientsafety/safesurgery/pulse_oximetry/who_ps_pulse_oxymetry_training_manual_en.pdf

